# B Chromosomes in Populations of Mammals Revisited

**DOI:** 10.3390/genes9100487

**Published:** 2018-10-09

**Authors:** Mladen Vujošević, Marija Rajičić, Jelena Blagojević

**Affiliations:** Institute for Biological Research “Siniša Stanković”, Department of Genetic Research, University of Belgrade, Bulevar despota Stefana 142, Belgrade 11060, Serbia; marija.rajicic@ibiss.bg.ac.rs (M.R.); jelena.blagojevic@ibiss.bg.ac.rs (J.B.)

**Keywords:** supernumerary chromosomes, additional chromosomes, chromosome polymorphism, evolution

## Abstract

The study of B chromosomes (Bs) started more than a century ago, while their presence in mammals dates since 1965. As the past two decades have seen huge progress in application of molecular techniques, we decided to throw a glance on new data on Bs in mammals and to review them. We listed 85 mammals with Bs that make 1.94% of karyotypically studied species. Contrary to general view, a typical B chromosome in mammals appears both as sub- or metacentric that is the same size as small chromosomes of standard complement. Both karyotypically stable and unstable species possess Bs. The presence of Bs in certain species influences the cell division, the degree of recombination, the development, a number of quantitative characteristics, the host-parasite interactions and their behaviour. There is at least some data on molecular structure of Bs recorded in nearly a quarter of species. Nevertheless, a more detailed molecular composition of Bs presently known for six mammalian species, confirms the presence of protein coding genes, and the transcriptional activity for some of them. Therefore, the idea that Bs are inert is outdated, but the role of Bs is yet to be determined. The maintenance of Bs is obviously not the same for all species, so the current models must be adapted while bearing in mind that Bs are not inactive as it was once thought.

## 1. Introduction

The presence of supernumerary or B chromosomes (Bs) is the oldest known chromosome polymorphism [1], and yet, after more than a century of research, the biological importance of Bs is still to be better determined. The knowledge about Bs in mammals is more recent and dates since 1965 when they were found in the greater glider, *Petauroides* (*Schoinobates*) *volans* by Hayman and Martin [2] and in the red fox, *Vulpes vulpes* by Moore and Elder [3].

A complex collection of diverse chromosomes, such as Bs, is difficult to describe. Yet, Bs are defined as dispensable supernumerary chromosomes which do not recombine with members of the basic A chromosome set (As), and do not follow the rules of Mendelian segregation law [4]. This definition assembles a pool of various chromosomes that do not share a complete set of features but only the mentioned dispensability, which alludes that a regular growth and development take place with or without Bs. A typical B chromosome is seen as a supernumerary, heterochromatic chromosome, smaller and morphologically different from chromosomes of the standard set, that does not evoke visible phenotypic effects. Nevertheless, the Bs that do not fit either partly or entirely into this picture are far from being atypical. In reality, when it comes to Bs, being out of the ordinary is considered to be a rule.

The earlier Beukeboom’s estimate that 15% of all species carry Bs seems to be too high. The more accurate calculation stating that only 3% of karyologically studied extant species, across the majority of taxonomic groups carry Bs, was given by D’Ambrosio et al. [5]. Although it was thought that species with Bs in mammals are many times less frequent than in plants, it seems that this is not well grounded. According to the data that Jones [6] summarized, there are 1252 plant species with Bs that make about 2.4% of karyotypically studied plant species [7]. In the first review of Bs in mammals Volobujev [8] listed 14 species, but the next year he expanded list to 25 species [9]. Vujošević [10] increased the list to 34 species, and in 2004 we recorded fifty-five species carrying Bs [11]. There are nearly 70 species with Bs that were mentioned by Trifonov et al. [12], but the very list of species was not presented. As it can be seen in Table 1, the number of mammalian species carrying Bs has increased to 85. At the same time, the list of documented mammalian species has also increased from 4629 [13] to 6399 extant ones [14]. It appears that ~1.9% of 4380 karyotypically studied mammalian species (according to chromosome number database [15]) are featured by presence of Bs. We gave all species proper names according to the list of Burgin et al. [14], but even in such a detailed list, some species remain questionable. Besides adding new species to the list, we also removed some due to either being listed multiple times under different names, or incorrectly mentioned as species with Bs, such as the pocket gopher, *Thomomys umbrinus* [16]. 

Despite the vast body of knowledge on Bs within mammalian species, the question of what factors determine the distribution of Bs across different species is yet to be answered. Why are Bs present in some species and not in others? Is there some innate property of the genome, or karyotype, which determines whether a species is likely to carry Bs or not? As passed two decades witnessed huge progress in application of molecular techniques, we decided to re-examine the data on Bs in mammals, and to suggest the future directions of the research.

## 2. Morphological Characteristics and Size of B Chromosomes

B chromosomes were sorted in three categories [8,10,11] based on their size in relation to chromosomes from the standard set (Table 1). The most frequent Bs are of the same size (II) as the chromosomes from A set (52 species, 65.0%), so they cannot be recognized using standard cytogenetic techniques. In this category, the size of Bs often corresponds to the size of the smallest chromosome in the genome. Furthermore, about less than half (39 species, 48.8%) of species have Bs that are smaller than the smallest chromosomes of the standard set. They belong to category I. Ungulates and bats have only Bs from this category (Table 1). There are two primate species that possess micro Bs, but there is still a debate if human small supernumerary marker chromosomes (sSMC) could be considered as B chromosomes [17].

The rarest (3.5%) of the species are the ones with Bs either larger or the same size as the largest chromosomes from the standard complement (III): *Uromys caudimaculatus* [18], *Holochilus brasiliensis* [19] and *Apodemus peninsulae* [20]. Additionally, the presence of different types of Bs in the same genome contributes to a large variability of Bs. Eleven species from Table 1 show variation in size and morphology of Bs, so there are different types that are recognised. This variation is well studied in *A. peninsulae* where five classes with different morphological types of Bs are present. 

Among Bs from type II, metacentrics and submetacentrics are more present (62.7%) than acrocentrics. Hewitt [21] noted that large Bs tend to be mitotically stabile while the small ones have an opposite tendency. It means that the intraindividual variability rises with the decrease in size of Bs (see more details in Section 3). 

## 3. Frequency of B Chromosomes

A large variability of Bs in mammalian species is displayed on all levels: intra-individual, intra- and inter-populational. The most common for mammals is frequent appearance of intraindividual variability that can feature the same tissue or appear between different tissues. Mosaicism for the number of Bs was scored in *Echymipera kalubu* [22], *Rattus rattus* [103], *V. vulpes* [117], *Myotis macrodactylus* [28], *A. peninsulae* [118], *Dicrostonix torquatus* [119], *Trinomys iheringi* [113], *Nictereutes procionides viverinus*, *Capreolus pygargus* [44], *Alouatta seniculus* [32], *Dasyprocta fuliginosa*, *Dasyprocta leporine*, *Dasyprocta prymnolopha* [72], *Apodemus flavicollis* [11], *Nictereutes procionides procyonides* [120], *Mazama nana* [51], *Mazama americana* [51], *Grammomys macmillani* [78], *Acomys ngurui*, *Tscherskia triton* [121] and *Mazama nemorivaga* [52]. 

The mosaicism for the number of Bs is extensively studied in Korean field mouse, *A. peninsulae*, first noticed in early studies [122] and then confirmed in different areas of species’ wide distribution [123,124]. The frequency of mosaics extends from 0.05 in South Korea [125] to even 0.85 in populations from Primorskii region and Hokkaido [123]. Furthermore, it has been found that the variability of B chromosome numbers is higher in the group of mosaics [8,124,126].

The great variability produced by intraindividual mosaicism is also characteristic for genus *Mazama*. In this genus, Bs appear in five out of eight species [51]. For instance, in *M. americana*, Abril et al. [127] found Bs in all 18 studied animals with intraindividual variability from 0–6 Bs. The same occurrence happened in *M. nana* [51] and in *M. nemorivaga* [52] where all studied animals had 0–7 Bs. Besides Bs, genus *Mazama* is featured with other kinds of chromosome polymorphisms, involving autosomes and sex chromosomes. This is also the case with *Acodon montensis* [128]. On the other hand, genus *Apodemus* with one third of species with Bs is karyotypically very stable.

A presence of one B chromosome is the most common situation, but the number of Bs per animal can vary widely. The highest number of Bs, which counted 42, was found in *D. torquatus* [75]. Up to 30 Bs in a single animal have been detected in *A. peninsulae* [65], while there have been 17 Bs identified in *Thamnomys gazellae* (now *Grammomys macmillani*) [78]. The average maximal number of Bs per specimen found in 85 mammalian species is 4.88 (Table 1).

There are some species with Bs whose populations cover wide geographic areas. The yellow-necked field mouse, *A. flavicollis*, common in the Western Palearctic region, has showed Bs presence almost everywhere through its range with frequencies ranging from 0.07 to 0.94 [62,129,130,131,132,133,134,135,136,137,138]. The frequency of animals with Bs in different geographic regions varies, but rules cannot be established easily. The variation in frequency of Bs that is generally present in *A. flavicollis* is also characteristic for small areas. For instance, we studied Bs presence in 40 populations from Serbia and the frequencies ranged from 0.11 to 0.67 [66,133,139,140,141,142]. Generally, the frequency of animals with Bs increases with altitude towards harsher climatological conditions [141,143]. However, this trend was not confirmed in the samples from Poland [138].

Shellhammer [144] suggested that the most reasonable explanation of great B frequency variation in southern marsh harvest mouse, *Reithrodontomys megalotis*, is a general increase in genetic variability towards the periphery of species distribution. The same was proposed for Bailey’s pocket mouse, *Chaetodipus baileyi* [70], while Boyeskorov et al. [145] found the highest B frequency in *A. flavicollis* (0.81) in a peripheral area of its distribution. A north-to-south increase in frequency of Bs was found in grassland Melomys, *Melomys burtoni* [85].

Besides being found in almost all studied populations, Bs in *A. peninsulae* are often present in all individuals. For instance, in the populations in East Asia, the frequency of animals with Bs vary from 0 to 1.0, while in the Siberian populations, from 0.99 to 1.0 [123,146]. So far, the only exceptions are Sakhalin Island and Stenina Island, where Bs repeatedly have not been found [146,147]. The distribution of Bs varies significantly between populations [62,148,149,150,151,152], however, these differences are still largely unexplained. The difference in the maximal number of Bs between regions is also evident varying from 30 in Siberia [65], to 6 in South Korea [123]. Roslik and Kartavtseva [124] established variability in modal number of Bs. Each population is characterized by a certain modal number of Bs. This number is also specific for regions. Roslik and Kartavtseva [153] documented the presence of clinal decreasing in frequency of rare B morphotypes from East to Northwest in the studied area.

Contrary to such high frequencies of Bs in *A. peninsulae* and *A. flavicollis*, Zima and Macholán [62] found that the frequency of animals with Bs in populations of long-tailed field mouse, *A. sylvaticus*, is very low (2.4%). Such sporadic occurrence of Bs is characteristic for another species from the same genus, the striped field mouse, *Apodemus agrarius* [59]. While *A. peninsulae* and *A. flavicollis* are typical forest-dwelling species, *A. sylvaticus* is limited to the edges of forests and *A. agrarius* is a typical field mouse.

## 4. Structure and Composition of B Chromosomes

The newly discovered facts about Bs are mostly concerning their structure. Bs were for a long time seen as chromosomes without genes or, at least without active ones, due to a prevailing absence of their visible phenotypic effects. Although the counterevidence was repeatedly suggested, they were generally ignored until recently when technological advances [154] in genome analysis and sequencing armed investigators with a variety of new technical approaches to shake this dogmatic view. Molecular studies represent Bs as assemblage of various repeated sequences originating from one or more A chromosomes [155,156,157] or even from all [158]. Non-coding repetitive sequences or mobile elements present in both A and B chromosomes prevail but some of them are more frequent in Bs [159]. Some paralogs of genes located on A chromosomes could be found on Bs as intact or as degenerate sequences [154]. Unique sequences specific for Bs are rarely found [23,160,161]. Yet, thanks to the new technology, the list of genes identified on Bs is promisingly increasing. 

The previous studies on mammalian Bs that were based mostly on differential staining revealed that 60% of them are C positive [11]. Those studies showed that when different types of Bs are present they could be C positive or C negative, such as in *A. peninsulae* and *M. nana* [51,162]. Furthermore, the analyses of molecular DNA composition of Bs in *A. peninsulae* [163] showed a presence of two specific forms of chromatin with presumed autonomous origin. Besides that, homology to the heterochromatic region of sex chromosomes and pericentromeric DNA of autosomes was established [163,164,165]. 

Molecular composition of Bs for 19 mammalian species is presented in Table 2. A presence of ribosomal genes (rDNA) was detected in 5 species by using silver staining and fluorescent in situ hybridization (FISH). Telomeric repeats are most frequently found on Bs (12 species), but centromeric were detected in only three cases. The presence of molecular markers specific for Bs was found in *P. volans* [23] and *A. flavicollis* [160].

The first autosomal gene found on Bs of mammals was proto-oncogene receptor tyrosine kinase (*C-KIT*). It was found in three unrelated species, the red fox, *V. vulpes*, the Chinese and Japanese raccoon dog, *N. procyonoides* [168,169] and *M. gouazoubira* [175] but not in *A. flavicollis* [179]. Another gene (*Vrk1*) was found in two *Apodemus* species. By using ISSR-PCR, Bugarski-Stanojević et al. [180] found a part of *Vrk1* gene on Bs of *A. flavicollis*. The presence of this gene was confirmed upon isolation by microdissection [181] and additional 37 genes or parts of genes were found on Bs of this species. The Bs in *A. flavicollis* have similar structure as pericentromeric region of sex chromosomes [188]. Through comparison of gene groups in Bs of six mammalian species from different families, Makunin et al., [173] confirmed enrichment with genes related to cell-cycle, development and genes functioning in the neuron synapse. They pointed that the presence of genes on Bs involved in cell-cycle regulation and tissue differentiation could be important for the B chromosome formation. 

There are also findings that propose the existence of regulatory interactions between coding sequences of A and B-chromosomes. Bugno-Poniewierska et al. [189], from studies of Bs in Chinese raccoon dogs and red fox determined that DNA methylation may maintain the transcriptional inactivation of DNA sequences situated on Bs. This could be the way to avoid some negative effects of Bs presence. Trifonov et al. [174] found, for the first time, the protein coding sequences on Bs of the Siberian roe deer, *C. pygargus*, which are not fully inactivated. Earlier, the gene expression in *A. flavicollis* showed elevated expression of three DNA fragments in the presence of Bs [178]. So, B chromosome could be seen as a repository of various information which could be used depending on the selection pressure that a B carrier faces. 

## 5. Origin of Bs in Mammals

There are several hypotheses proposed to explain the route of B chromosomes appearance [174,185,190]. In general, the source of Bs are chromosomes of the standard set, both autosomes and sex chromosomes, yet their origin from interspecies hybrids has also been proven in certain cases [191,192], but not in mammals. Whatever the source of their origin is, all proto-Bs must instantaneously pass through inactivation to avoid synapsis with the source chromosome. At present, a series of molecular processes are known as good candidates to achieve this condition, for instance mechanisms of sex chromosomes inactivation and epigenetic mechanisms. Bs can follow the same process operating in meiotic sex chromosome inactivation (MSCI) during the meiotic prophase I. Vujošević and Blagojević [11] proposed that B chromosomes are absent in birds due to genome reduction. Moreover, it appears that the sex chromosome specific silencing is absent in birds, although not yet been completely elucidated [193,194]. The same situation is found in egg laying monotremes [195] that also lack Bs. What is frequently overlooked in attempts to explain the initial steps of Bs origin is the possibility of simultaneous origin of proto-Bs in a population [11] that is far more probable in mammals due to their social organisation and population dynamics. This could promote spread of proto-Bs in populations.

The origin of Bs in mammalian species was based rather on presumptions than on facts. The circumstantial evidence come and is expected from molecular studies of Bs DNA contant. Sex chromosomes are proposed as a source of Bs in *E. kalubu* [22], *Dycrostonix groenlandicus* [196] and *Apodemus argenteus* [60]. In *A. peninsulae* a homology of heterochromatic region of Bs, sex chromosomes and autosomes was established [163,164,165]. Upon generation of microdissected DNA probes followed by FISH on metaphase chromosomes, another study found that Bs in *A. flavicollis* originate from pericentromeric region of sex chromosomes [188]. While there are five different types of Bs [197] with different origin (including sex chromosomes) in *A. peninsulae*, it was shown that all Bs in *A. flavicollis* have the same DNA contant regardless of their number or geographical distance which indicates a common origin from sex chromosomes [188]. Furthermore, whenever two or more types of Bs are present in one species, it appears that they do not have the same origin. A multiple origin of Bs in *A. peninsulae* was suggested by Matsubara et al., [198] based on the presence of 18S/28S rRNA genes only on meta- or submetacentric Bs. Some recent findings offer evidences for single origin of Bs in this species also [181]. A different origin for two types of Bs was also found in the harvest mouse, *R. megalotis*, by Peppers et al. [187].

Based on comparative cytogenetic studies [67,199] it was suggested that Bs in *Bandicota indica*, *R. rattus* and *Rattus fuscipes* originated before the divergence of these species occurred. Different origin was assumed for Bs in two species of Carnivora, *N. procyonides* and *V. vulpes* [163,173] based on molecular data. 

A two-step appearance of Bs was proposed for *A. peninsulae* [164]. The first step is the destabilization of pericentromeric regions, produced by the invasion of DNA sequences from euchromatic parts of A chromosomes, which leads to a formation of microchromosomes in high frequency, and thus make proto-Bs. The second step is the insertion and amplification of new DNA sequences. Similar steps were proposed by Rubtsov et al. [200] that assumed that the origin of Bs start with a loss of a greater part of q arm of an ancestor autosome followed by subsequent evolution of Bs that includes additional constitutional rearrangements. Makunin et al. [175], by using sequencing of isolated Bs of two mammalian species, showed that Bs originate as segmental duplications of specific genomic regions, and subsequently passes through pseudogenization and a repeat accumulation.

Presently, it seems that the new data describing the molecular composition of Bs incites more questions than suggests answers to the old ones.

## 6. Behaviour of B Chromosomes during Meiosis and their Transmission

The number of species whose meiotic behaviour of Bs was studied increased just slightely in last 15 years but there are new details for some already studied species. Currently, the meiotic behaviour is known for 25 species and univalent Bs are present in all of them. Besides univalents, bivalents appear in 13 species, and multivalents in 7, while assimetrical bivalents are present in 6 species. There are 5 species (*A. peninsulae*, *D. groenlandicus*, *N. procyonides*, *C. baileyi* and *V. vulpes*) where all four mentioned types of configurations are found.

When a different type of Bs is characteristic for the same species, their meiotic behaviour is often type dependent. So Hyata [64] found that both paring and non-paring among Bs occur in *A. peninsulae*. He showed that small macro- and microchromosomes in most cases do not follow Mendelian inheritance, yet other types of supernumeraries do follow it. Further meiotic studies in this species [197] showed that Bs are able to form axial elements and synaptonemal complexes in prophase of the first meiotic division. The same authors found that univalents of dot like Bs of different morphology are obviously not homologous, while metacentric Bs showed a partial homology. Univalent Bs are commonly associated with sex bivalent. Ishak et al. [201] noticed an absence of transcriptional activity in Bs of this species during pachytene. Karamysheva et al. [202], through the use of 2D analysis of pachytene in *A. peninsulae*, found three types of configurations: synapsed bivalents, univalents, and univalents that contain the foldback structure. During meiosis, Bs in *A. flavicollis* appear as univalents, bivalents or, depending on number, combinations of both, but never as a pair with the members of A set [130,203]. In the same species, Banaszek and Jadwiszczak [204] found that Bs behave in non-Mendelian fashion during meiosis I of males.

In *N. squamipes* analysis of the synaptonemal complex revealed auto-pairing of univalent Bs [184]. In the Northen collared lemming, *D. groenlandicus*, it was found that, besides univalents, bivalents and trivalents Bs can make synaptic associations with the Y chromosome [196]. Studies of synaptonemal complex in *M. americana* by Aquino el al. [46] revealed the presence of both univalents and bivalents. Univalents appear in two forms: as autopaird or just univalents. 

When Bs appear as univalents in the silver fox, they show a folding-back behaviour that ends as intrachromosomal pairing [205], which indicates the presence of repeated DNA sequences. Sosnowski et al. [206] conducted experiments with spermatocytes of the red fox and the Chinese raccoon dog, and found that Bs that conjugate together form diverse structures, such as bivalents, trivalents, and tetravalents. Sosnowski et al. [206] also concluded that the increase in the number of Bs in spermatocytes of the Chinese raccoon dog corresponds with the lack of conjugation more frequent. Basheva et al. [43] studied A- and B-chromosome pairing and recombination in the silver fox using electron and immunofluorescent microscopy. They found the same distribution of the foci along B- and A-bivalents and proved, for the first time, that meiotic recombination occurs in mammalian B chromosomes

The accumulation of B chromosomes in mammals appears to be a rarer event than expected. One of the reasons for sure is the lack of studies. Furthermore, Bs in some cases could maintain themselves without the apparent drive. In males, the evidences for accumulation of Bs were found in *C. baileyi* [207], *V. vulpes* [208], *A. peninsulae* [162,197,209], and in the greater long tailed hamster, *T. triton* [121]. In the latter, Bs were found in males only and the increase in number of Bs in germline cells was observed. 

In lemmings, univalent Bs were eliminated from the polar body and incorporated into secondary oocytes [210,211]. The evidence of accumulation of Bs has been obtained in females of *R. rattus* [212,213] and *R. fuscipes* [97] by means of controlled crosses. In the case of experimental crosses done by Stitou et al. [213] in *R. rattus*, males showed Mendelian transmission rates, while only a slight accumulation of Bs happened in females.

Palestis et al. [214], following the theory of centromeric drive, based on a different ability of the two meiotic poles for capturing centromeres [215], showed that Bs in mammals are more common in species with acrocentric chromosomes. Since then, the number of mammalian species with Bs increased, therefore this theory is not valid anymore. The number of species with Bs with predominantly acrocentric chromosomes in standard set is just slightly larger, so it seems that such explanation for origin of Bs is reasonable only in proven cases.

Karamysheva et al. [202] studied nuclear organization of Bs in *A. peninsulae*. They showed that additional volume of heterochromatic regions of chromosomes and extra centromeres modify 3D architecture of interphase nuclei. The location of Bs in meiosis appeared not to be random, and unpaired Bs had a tendency to form a common compartment with unpaired part of the sex bivalent, and thus avoided pachytene check point.

## 7. The Effects of B-Chromosomes

Apart from few exceptions, Bs do not cause visible phenotypic manifestations at individual level. This makes the search for observable effects in mammals rather difficult. But even such a small amount of data, together with new findings of genes on Bs, raises objections to the idea of Bs genetic inertness. It has been found that Bs presence influences cell division, degree of recombination, development, some quantitative characteristics, host-parasite interactions and behaviour.

A new and interesting data came from three-dimensional studies of Bs behaviour during division in both somatic and germ cells. Kociucka et al. [216] studied three-dimensional positioning of B chromosomes in fibroblast nuclei of the red fox and the Chinese raccoon dog, and found that small Bs of the red fox are dominantly positioned in the interior of the nucleus, while the medium-sized Bs of the Chinese raccoon dog are in the peripheral area of the nucleus as well as in intermediate and interior locations. The data was in agreement with the chromosome size dependent theory [216]. But in the nuclei of the Korean field mouse all Bs, irrespective of their size, were located on nuclear periphery in common compartments with C-positive regions of A chromosomes [202]. They suppose that, at least for the Bs of the Korean field mouse, the DNA content is more important parameter that determines where Bs will be located inside the nucleus. 

As we pointed earlier, Basheva et al. [43] proved that in the silver fox recombination occurs between B chromosomes which increases variability in the specimens that carry them. In earlier studies, the presence of B chromosomes was associated with increased chiasma frequency in Bailey’s pocket mouse, *C. baileyi* [207] and *R. fuscipes* [199]. In both species this increase is not influenced by the number of Bs yet only by their presence.

Gileva [211] recorded a reduction of body and skull sizes in *D. torquatus* that carry numerous Bs, and proposed that this could be reflected as a negative selective value in extreme climate conditions. Positive correlations between number of Bs and body weight were established in males of two species: *N. p. viverinus* [217] and *A. flavicollis* [62,218]. Effects of Bs presence are extensively studied in *A. flavicollis* and, in general, it was found that they influence the development of some morphometric characters, mostly cranial ones [140]. One of the two regions of the mandible shows almost a triple increase in intensity of integration in B carriers [219]. Furthermore, the maintenance of Bs in the same species was studied by examining their effects on 3 components of cranial variability: canalization, developmental stability, and morphological integration. It was suggested that B carriers follow different developmental pathway for generating covariations of cranial traits [220]. This specific developmental pathway is more sensitive to modifications caused by natural selection, which could be beneficial to B carriers under variable environmental conditions. It was previously established that reaction of animals with Bs to environmental changes differ from those without them [221]. Nonmetric traits analyses show that the population density influences, at the same time, both the variation in the frequency of specimens with Bs and the developmental homeostasis.

Adnađević et al. [222], by analysing effect of recorded endoparasites and parasite life-cycle stages in *A. flavicollis* on expression levels of genes *MHC II-DRB*, *IL-10* and *Tgf-β*, found that the presence of Bs is associated with lower expression level of *Tgf-β* gene. Although the influence of host genetic background on parasite infection has already been well established, this is the first study in mammals that correlates presence of Bs with immune response. Curiously enough, the presence of Bs in this species plays an important role in infrapopulations of their certain endoparasites by shifting sex balance to higher proportion of males [223].

Shellhammer [144] from studies on *R. megalotis* was the first to propose that Bs could have an effect on behaviour. The behaviour and the presence of Bs were connected in foxes through a series of experimental crosses [224,225,226]. It appeared that groups of foxes selected for specific behaviour differs significantly in frequency of mosaics for Bs.

## 8. Maintenance of B Chromosomes

The mostly discussed question about Bs during a century of research was the way they are maintained in populations through time. Two schools of thought grouped around two models giving opposite explanations of the way Bs are retained in natural populations. Both models assume that the frequency of specimens with Bs in population is at equilibrium but the explanations how this equilibrium is reached and kept are different. The model firstly named parasitic and then selfish [227] claims that Bs are maintained by balance of accumulation and elimination due to detrimental effects. Contrary to this, the heterotic model [228] suggests that, in the absence of mechanism of accumulation, a small number of Bs could offer an adaptive advantage to carriers, while a large number could be harmful. Currently, the parasitic model is predominant, mostly because the search for adaptive significance of Bs was mostly ineffective. Furthermore, the convergence of this paradigm partly comes from the popular theory of selfish or parasitic DNA, irresistible to some scientists. The number of cases with proved Bs accumulation, which is prerequisite for parasitic model, although larger than the number of cases without accumulation, is still very small in comparison with the known number of species with Bs. For instance, the accumulation of Bs was studied in about 70 plant species and among them 42 (60%) manifested accumulation mechanism [4] which makes only 3.4% of plant species with Bs. Furthermore, detailed studies are largely directed on commercially important species that possess Bs, like maize and rye, and pests such as grasshoppers, so the number of extensively studied species groups is rather small. In attempt to include species without mechanism of Bs accumulation into parasitic model, Camacho et al. [229] proposed that all Bs are initially parasitic, and later on, through arm race with A genome, may become neutral. From this stage they can disappear or become parasitic again. One of their arguments against heterotic Bs is that it is unexpected that Bs could be beneficial in the first step, so a drive is necessary to establish themselves in population. Yet, if Bs appear simultaneously in population, these arguments are not plausible. Therefore, when models are assessed it is not good to stay frozen within a particular paradigm.

Temporal analyses of B frequency and transmission in mammals are scarce. The frequency of animals with Bs was the same in two successive years in *Rattus rattus diardi* [100]. During 8 years of study, equilibrium frequencies of Bs in populations of *A. flavicollis* at one locality were maintained in spite of fluctuations in population density [11,230]. Zima and Macholan [62] and Wojcik et al. [138] also found the equilibrium during a three-year study in the same species. Contrary to stable frequency from year to year, seasonal changes in frequency of animals with Bs could escape from equilibrium in stress situations [231] or could keep it when there is no tough competition present [232]. Thus, though frequencies of Bs could significantly differ through a year, their values stay the same between years [230]. 

B chromosome frequencies in *A. peninsulae* show temporal variation. Comparison of Bs from the population from Altai Republic, trapped in the 1980 and 2002 showed that a mean number of Bs in this population increased almost threefold in the period of 22 years [200]. This increase was mainly due to the rise of numbers of small and large bi-armed Bs (by factors of 7.0 and 5.3, respectively) and a slight increase in the number of medium-sized biarmed B chromosomes (by a factor of 1.6). Nonetheless, Borisov et al. [233] found that the number of Bs and theirs morphotypes were stable over the period of 30 years in certain populations.

Direct or indirect evidences for B drive in mammals are provided for seven species only: *C. baileyi* [208], *V. vulpes* [209], *R. rattus* [212,213], *D. torquatus* [211], *R. fuscipes* [97], *A. peninsulae* [197], and *T. triton* [121]. In 3 of them, B drive is operating in females thus supporting the theory of centromeric drive [214]. Thomson [97] showed that the maintenance of Bs in *R. fuscipes* supports the parasitic model very well.

The maintenance of Bs in populations can be explained in terms of their contribution to overall genetic diversity of the species possessing them, and it might be arguable under the heterotic model [142]. The increased variability widens the probability that species will survive in changing environmental situations. In *A. flavicollis*, an increased frequency of animals with Bs is found in more extreme climatic conditions [141]. Frequency of B chromosomes and quality of habitat are negatively correlated indicating that B chromosomes in this species are mentioned due to the effects that they exert at the level of populations [143]. Possible adaptive effects of Bs were also postulated by Blagojević et al. [234] upon comparison of head morphology in three populations of this species that have Bs at different frequencies. Adnađević et al. [235], by using amplified fragment length polymorphism (AFLP) markers, made a comparison of populations of *A. flavicollis* settled in ecologically distinct habitats differing in frequency of Bs, and found that the greatest genetic diversity is in the population settled in optimal conditions for this species featured by the lowest frequency of animals with Bs. The majority of loci that are subject of directional selection, feature either population with lower or with a higher frequency of Bs. They suggested that the different frequency of B carriers in populations is related to adaptive differentiation to diverse habitats. Tokarskaia et al. [45] found that the presence of Bs is positively correlated with heterozygosity for random amplification of polymorphic DNA (RAPD) loci, in populations of the Siberian roe deer, *C. pygargus*, thus indicating influence of Bs on the genetic variation of the species. All these findings support the heterotic model of Bs maintenance.

Theoretically, inbreeding is harmful to parasitic Bs but beneficial to mutualistic ones. Social organization of rodent populations and some other mammalian groups supports inbreeding which opens new possibilities for the existence of beneficial Bs.

Extensive population studies of two species of the same genus *Apodemus* best illustrate that the present models do not exclude each other but rather call for further adjustments. If we try to fit the maintenance of Bs in *A. peninsulae* and *A. flavicollis* into the current models, *A. peninsulae* will follow the parasitic (or selfish) model, while Bs in *A. flavicollis* will better fit into heterotic model. But when we go into details, it seems that neither *A. peninsulae* nor *A. flavicollis* fit quite well into proposed models. *A. peninsule* do not have populations at equilibrium and tolerance for Bs is so great that it is not easy to say when Bs become detrimental. Furthermore, five different types of Bs present in this species, have different outcomes. Some types are inherited in almost a Mendelian fashion. On the other hand, Bs in *A. flavicollis* brings adaptive advantage in certain situations and in some environments. In other situations (and environments) they could be neutral or deleterious. Therefore, it could be hypothesized that the adaptive advantage of these Bs is not general, but it is dependent on events through which the individual or population is passing. The existing models need to be very much adjusted, but the adjustment must be based on detailed and intensive studies in natural populations. 

## 9. Conclusions

After more than a century, it appears that B chromosomes research suffers from an unbalanced approach. That is also true for research of Bs in mammals. Population studies are a very difficult task and are still largely avoided. Even rarer attempts are made to resolve effects of Bs in different species that carry them. While molecular breaks into DNA composition of Bs are rapidly increasing, the number of species included in them is still scarce. Namely, a more detailed molecular composition is known for only six mammalian species. Although the confirmed presence of genes on Bs, in all cases, disproved the claims that Bs are inert, the gathered knowledge and data are not sufficient to explain the significance of Bs to their carriers. Are the paths of evolution of As and Bs opposite, or do the lanes of the same highway promise a greater success in adapting to environmental changes? This is yet to be resolved, but the answer seems to be inclining towards the latter statement.

## Figures and Tables

**Table 1:** List of species with B chromosomes.

ORDER *Species*	Common Name ^(◊)^	2n	NFa	X/Y	No. Bs	Bs Morphology		References
						Size *	Cent. Position ^†^	
**PERAMELEMORPHIA**								
*Echymipera kalubu*	Common Echymipera	13–14 XX/X0	26	M/A	1–5	I	M	[22]
**DIPROTODONTIA**								
*Petauroides (Schoinobates) volans*	Greater glider	22	38	M/A	1–8	I	mi	[2,23]
**INSECTIVORA**								
*Crocidura leucodon*	Bicolored shrew	28	52	SM/SM, A	1	II	A	[24]
*Crocidura malayana*	Malayan shrew	38	62	SM/M	1–2	II	M	[25]
*Crocidura suaveolens*	Lesser shrew	40	46	M/A	1	II		[26]
*Sorex bedfordiae*	Lesser stripe-backed shrew	24	44	A/A	1–2	II	M	[27]
**CHIROPTERA**								
*Myotis macrodactylus*	Big-footed Myotis	44	56	M/A	1	I	mi	[28]
*Nyctalus leisleri*	Lesser Noctule	44	54	М/А	1–3	I	mi	[29]
*Pipistrellus tenuis (mimus)*	Least Pipistrelle	38	50	М/А	2–4	I	mi	[30]
**PRIMATES**								
*Alouatta seniculus* *A.seniculus macconelli*	Red howler monkey	46 47–49	64	M/A	1–3 1–3	I	A	[31,32] [33]
*Homo sapiens*	Human	46	78	SM/A	2	I	mi	[34,35]
**CARNIVORA**								
*Atelocynus microtis*	Short-eared dog	74	72	SM/SM	2	I	mi	[36]
*Chrysocyon brachyurus*	Maned wolf	76	72	SM/SM	1	II	A	[37]
*Nyctereutes p. procyonoides*	Raccoon dog	54	62	M/M	1–4	II	A, SM	[38]
*Nyctereutes p. viverinus*		38	62	M/M	1–5	II	A	[39]
*Vulpes (Alopex) lagopus*	Arctic fox	50	92	M/A	1	II	M	[40]
*Vulpes bengalensis*	Bengal fox	60	68	M/A		I	mi	[41]
*Vulpes pallida*	Pale fox							[42]
*Vulpes vulpes (fulvus)*	Red fox	34	64	M/A	1–10	I	A, M	[3,43]
**ARTIODACTYLA**								
*Capreolus pygargus*	Siberian roe deer	70	72	SM/A	1–14	I	mi	[44,45]
*Mazama americana*	Red brocket	42–53	42–52	SM/A	2–5	I	mi, A	[44,46]
*Mazama bororo*	Small red brocket	34	46	M	4–6	I	mi, A	[47,48]
*Mazama gouazoubira*	Gray brocket	69–70 70	68–69 68	M, A/A, mi A/M	1–2 1–3	I I	mi, A	[47,49,50]
*Mazama nana*	Brazilian dwarf brocket	36	54	M/mi	1–6	I	mi, A	[47,51]
*Mazama nemorivaga*	Amazonian brown brocket	67–69	69–72	SM/A,M	2–7	I	mi	[52]
*Moschus moschiferus (sibiricus)*	Siberian musk deer	58	56	A/A	1–2	-	-	[53]
**RODENTIA**								
*Acomys ngurui*		59–61	68	M/A,SM	1	II	SM	[54,55]
*Acomys spinosissimus*	Spiny mouse	59–61		A/SM	1	II	A	[54]
*Akodon mollis*	Soft grass mouse	22	42	M/A	1	II	M	[56]
*Akodon montensis (arviculoides)*	Montane Akodont	24	42	A/A	1–3	II	SM	[57,58]
*Apodemus agrarius*	Striped field mouse	48	54	А/А	1	I, II	mi, A	[59]
*Apodemus argenteus*	Small Japanese field mouse	46	48	A/A	1	I, II	mi, SM	[60]
*Apodemus flavicollis*	Yellow-necked field mouse	48	46	А/А	1–9	II	A	[61,62]
*Apodemus mystacinus*	Eastern broad-toothed field mouse	48	50	A/A	2	-	-	[63]
*Apodemus peninsulae (giliacus)*	Korean field mouse	48	46	A/A	1–30	I, II, III	mi, A, SM, M	[64,65]
*Apodemus sylvaticus*	Long-tailed field mouse	48	46	A/A	1–3	II	A	[66]
*Bandicota indica*	Greater bandicoot rat	44/45 XX/XO	84	SM/A	1–3	II	SM	[67]
*Bandicota savilei*	Savile’s bandicoot rat	43	58	SM	1	I	SM	[68]
*Berylmys berdmorei*	Berdmore’s Berylmys	40	62	A	1	II	M	[68]
*Blarinomys breviceps*	Brazilian shrew mouse	29–50	50	A	2	II	M	[69]
*Chaetodipus (Perognathus) baileyi*	Bailey’s pocket mouse	46	64	M/M	1–10	II	M	[70]
*Dasymys rufulus*	West African shaggy rat	36, 38 39, 40	42–50	A,SM, M/A, SM, M	1–3	II	M	[71]
*Dasyprocta fuliginosa*	Black Agouti	64	118	M/SM	1	II	SM	[72]
*Dasyprocta leporina*	Red-rumped Agouti	64	118	M/M	1	I	M	[72]
*Dasyprocta prymnolopha* (*nigriclunis*)	Black-rumped Agouti	64	118	M/SM	1	II	M	[72]
*Dasyprocta* sp.	-	64	118	M/SM	1	II	M	[72]
*Dicrostonyx groenlandicus (kilangmiutak)*	Northern collared lemming	48		M/SM	1–3	I, II	A, M	[73]
		47–50	48	A, M/A,SM	1–8	II	M	[74]
*Dicrostonyx torquatus*	Palearctic collared lemming	44	56	A, SM/A	1–42	II	SM, M	[74,75]
*Golunda ellioti*	Indian bush rat	54	54	SM/A	1–4	II	A	[76]
*Grammomys (Thamnomys) dolichurus*	Woodland thicket rat	54	68	SM/A	4-7	II	A, M	[77]
*Grammomys macmillani (Thamnomys gazellae)*	Macmillan’s thicket rat	54	70	SM/A	2–17	I	mi	[78]
*Holochilus brasiliensis*	Web-footed marsh rat	48	58	A/A	1–2	II, III	SM, M	[79]
*Holochilus chacarius*	Chaco marsh rat	48–56	56–60		1–2	II		[80]
*Holochilus venezuelae*	-	44	56	A/A	1	II	M	[81]
*Holochilus vulpinus*	-	36	58	A/A	1–3	II	A	[19]
*Mastacomys fuscus*	Broad-toothed rat	48	56	SM/SM	1	II	A	[82]
*Mastomys erythroleucus*	Guinea multimammate mouse	38	54	SM/SM	2	II	A	[83]
*Mastomys natalensis*	Natal multimammate mouse	32	54	M/A	1	II	SM	[84]
*Melomys burtoni*	Grassland Melomys	48	50	A/A	1–8	I, II	mi, A, SM, M	[85]
*Melomys capensis*	Cape York Melomys	48	50	A/A	3–6	-		[85]
*Melomys cervinipes*	Fawn-footed Melomys	48	50	A/A	4–13	I, II	SM, A	[82]
*Microtus gregalis*	Narrow-headed vole	36	50	M/A	1–4	II	A	[86]
*Microtus longicaudus*	San bernardino long-tailed vole	56	84	M/A	1–14	I	M	[87]
*Mus cookii*	Ryley’s spiny mouse	40	38	A/A	1	I, II	A, M	[68]
*Mus shortridgei*	Shortridge’s mouse	46	46	A/A	1–3	I, II	A, M	[88]
*Nannospalax (Spalax) leucodon*	Lesser blind mole rat	60	74	SM/M	1–3	I	mi, A	[89]
*Nectomys rattus*	Common water rat	52	50	A, SM/A, SM	1–3	II	A, SM, M	[90]
*Nectomys squamipes*	South American water rat	56	54	A, SM/A, SM, M	1–3	II	A, SM	[91]
*Oecomys concolor*	Natterer’s Oecomys	60	62		1–2	I	SM	[92]
*Oligoryzomys (Oryzomys) fornesi*	Fornes colilargo	62–66	64	SM/SM	1–2	I	A	[93]
*Oligoryzomys flavescens*	Yellow pygmy rice rat	64	64	SM/SM	1–2	I	mi	[94]
*Otomys irroratus*	Southern African vlei rat	28	44	M/SM	2–4	II	SM, M	[95]
*Proechimys* sp.		26			1	I	mi	[96]
*Rattus fuscipes*	Bush rat	38	58	A/A	1–3	II	M	[82,97]
*Rattus norvegicus*	Brown rat	42	60	A/A	1	II	A	[98]
*Rattus rattus*	House rat	42	60–64	A/A	1–3	II	M	[99]
*Rattus r. diardii*		42			1–4	II	M	[100]
*Rattus r. frugivorus*		38			1–3	II	M	[101]
*Rattus r. kandianus*		40			1	II	M	[102]
*Rattus r. tahnezumi*		42			1	II	M	[102]
*Rattus r. thai*		42			1-6			[103]
*Rattus tunneyi*	Pale field rat	42	60	A/A	1	II	M	[104]
*Reithrodontomys megalotis*	Southern marsh harvest mouse	42			1–7	I	mi	[105]
*Reithrodontomys montanus*	Plains harvest mouse	36	72	M/A, SM	1	I	SM	[106]
*Sigmodon hispidus*	Hispid cotton rat	52	50	A/M	3–4	-		[107]
*Sooretamys angouya (Oryzomys angouya, O. buccinatus, O. ratticeps)*		58	60	A/A	2	I, II	mi, SM	[108,109,110]
*Thallomys nigricauda*	Black-tailed tree rat	48	60			-		[111]
*Thomomys bottae*	Animas mountains pocket gopher	76	130	SM/mi	6–12	I	mi	[112]
*Trinomys (Proechimys) iheringi*	Ihering’s spiny rat	60	116	SM/SM	1–6	I	mi	[113,114]
*Tscherskia (Cricettilus) triton*	Greater long-tailed hamster	28	30	A/M	1–2	II	A	[112,113]
*Uromys caudimaculatus*	White-tailed giant rat	46	50	A/A	2–12	II, III	A, SM, M	[18,115]

2n—diploid number, NFa—fundamental number of autosomes, X/Y—morphology of sex chromosomes; * Category: I—Bs smaller than chromosomes from A set, II—same as A, III—larger than A; ^†^ mi—micro Bs, M—metacentric, SM—submetacentric, A—acrocentric chromosomes; (◊) Mammal Diversity Database [116].

**Table 2:** Current data on molecular composition of B chromosomes in mammalian species.

Species	Found on B Chromosome	Method	References
*Petauroides volans*	centromeric regions, B specific regions	FISH, PCR	[23]
*Nyctereutes procyonoides procyonoides*	interstitial telomeric sequences	FISH	[166]
	rDNA (NOR)	FISH, silver staining	[167]
	*C-KIT*	FISH	[168]
	*Kdr*, *RPL23A* pseudogene	FISH, PCR	[169]
	rDNA	PRINS (primed in situ DNA synthesis)	[170]
	*Lrig1*	FISH	[171]
	*Ret*		
	*Lrig1* *Ret*	FISH	
	*C-KIT* (no transcriptional activitiy)	PCR, RT-PCR	[172]
	100 sequences located on B, homologous to genes involved in cell proliferation, differentiation, neuron sinapse, cell junction	sequencing of microdissected B	[173]
*Nyctereutes procyonoides viverrinus*	interstitial telomeric sequences	FISH	[166]
	three types of B-specific heterochromatin	FISH	[173]
	*C-KIT*	FISH	[168,169,171]
	*Kdr* *RPL23A* pseudogene	FISH, PCR	[169]
*Vulpes vulpes*	*C-KIT*	FISH	[168,171]
	*RPL23A* pseudogene	PCR	[169]
	*Mdn1*, *Ctndd2*	FISH	[171]
	49 sequences located on B, homologous to genes associated with cell division machinery, cell cycle control functions, microtubule, centrosomes, cell differentiation, proliferation	sequencing of microdissected B	[173]
*Capreolus pygargus*	*Tnni3k*, *Fpgt*, *Lrriq3*	FISH, flow-sorted DNA libraries derived from Bs	[174,175]
	9 genes located on B	re-analyzed data from [175]	[173]
*Mazama gouazoubira*	55 sequences located on B, homologous to genes associated with functional clusters associated with ATP-binding/kinase, mitochondria, cell cycle, Zn-ion binding/Zn-finger, membrane, cell proliferation/ differentiation, positive regulation of protein kinase activity	sequencing of microdissected B	[175]
	107 sequences located on B homologous to genes	re-analyzed data from [175]	[173]
*Acomys* sp.	telomeric repeat	FISH	[54]
*Akodon montensis (arviculoides)*	rDNA (NOR)	silver staining	[57,108]
	telomeric repeat, rDNA (NOR)	FISH, silver staining	[176]
*Apodemus flavicollis*	rDNA (NOR)	silver staining	[177]
	B specific regions	AP-PCR RT-PCR	[160] [178]
	rDNA	RT-PCR	[179]
	*Vrk1*	ISSR-PCR, sequencing	[180]
	38 sequences located on B, homologous to genes associated with microtubule, cell cycle proteins, and less significant nucleotide-binding, membrane and metal binding proteins. Satellite repeats, MurSatRep1, ERVL (MaLR), ERVK LTRs and transposable elements.	sequencing of microdissected B	[181]
	101 sequences located on B homologous to genes	re-analyzed data from [181]	[173]
*Apodemus peninsulae*	telomeric repeat, two types of B arm-specific repeats	FISH	[164] [163]
	two types of B-specific chromatin	FISH	[181]
	repetitive elements	FISH	[182]
	centromeric repeats, 32 sequences located on B homologous to genes associated with cell division machinery, cell cycle control, nucleotide-binding, laminin and EGF-like domain-containing, cytoskeleton and ion-bindings proteins, LINE L1 elements, centromeric repeats, satelite repeats MurSatRep1, ERVK and ERVL (MaLR) LTRs.	sequencing of microdissected Bs	[181]
	152 sequencies located on Bs homologous to genes	re-analyzed data from [181]	[173]
*Blarinomys breviceps*	telomeric repeats, ITSs	FISH	[69]
*Holochilus brasilensis*	OSHR, telomeric repeats	FISH	[183]
*Nanospalax leucodon*	telomeric repeat	FISH	[89]
*Nectomys* sp.	ITBs	FISH	[184]
*Nectomys rattus*	OSHR	FISH	[183]
*Nectomys squamipes*	OSHR, ITS	FISH	[183]
*Rattus rattus*	rDNA	FISH	[185]
	telomeric repeat	FISH	[186]
*Reithrodontomys megalotis*	telomeric repeat, LINE elements, centromeric repeats	FISH	[187]
*Sooretamys angouya*	rDNA (NOR)	silver staining	[108]
*Trinomys iheringi*	telomeric repeats	FISH	[114]

FISH—fluorecent in situ hybridization; RT-PCR—real time-PCR; rDNA (NOR)—ribosomal DNA (nucleolus organizer region); AP-PCR—arbitrarily primed-PCR; ISSR-PCR—inter simple sequence repeat-PCR; EGF—epidermal growth factor; LINE—long interspersed nuclear element; ERVK—endogenous retrovirus-K; ERVL—endogenous retroviruses-related; LTRs—long terminal repeats; ITS—interstitial telomeric sequences; OSHR—Oryzomyini shared heterochromatin region; ITBs—interstitial telomeric bands.
